# Analyzing spatial aggregation error in statistical models of late-stage cancer risk: a Monte Carlo simulation approach

**DOI:** 10.1186/1476-072X-9-51

**Published:** 2010-10-19

**Authors:** Lan Luo, Sara McLafferty, Fahui Wang

**Affiliations:** 1 Department of Geography, University of Illinois at Urbana-Champaign, Room 220 Davenport Hall, 607 S. Mathews Ave, Urbana, IL, USA 61801-3671; 2 Department of Geography and Anthropology, Louisiana State University, Baton Rouge, LA, USA 70803

## Abstract

**Purpose:**

This paper examines the effect of spatial aggregation error on statistical estimates of the association between spatial access to health care and late-stage cancer.

**Methods:**

Monte Carlo simulation was used to disaggregate cancer cases for two Illinois counties from zip code to census block in proportion to the age-race composition of the block population. After the disaggregation, a hierarchical logistic model was estimated examining the relationship between late-stage breast cancer and risk factors including travel distance to mammography, at both the zip code and census block levels. Model coefficients were compared between the two levels to assess the impact of spatial aggregation error.

**Results:**

We found that spatial aggregation error influences the coefficients of regression-type models at the zip code level, and this impact is highly dependent on the study area. In one study area (Kane County), block-level coefficients were very similar to those estimated on the basis of zip code data; whereas in the other study area (Peoria County), the two sets of coefficients differed substantially raising the possibility of drawing inaccurate inferences about the association between distance to mammography and late-stage cancer risk.

**Conclusions:**

Spatial aggregation error can significantly affect the coefficient values and inferences drawn from statistical models of the association between cancer outcomes and spatial and non-spatial variables. Relying on data at the zip code level may lead to inaccurate findings on health risk factors.

## Introduction

Detecting and analyzing spatial aggregation error in large spatial data sets is an increasingly important topic in GIS and public health research [1-5]. Spatial aggregation error arises because of the agglomeration of individual, georeferenced observations into larger spatial zones. The spatial aggregation process smoothes local variation, leading to errors in measurement of geographical variables. This error in turn affects the estimation of statistical models that incorporate spatially-aggregated variables. Spatial aggregation error is particularly important in cancer research, given that cancer data sets are often only released publicly at the zip code level due to privacy and confidentiality issues[6]. Thus, studies that use zip code-level data to examine the associations between geographical and environmental variables and cancer incidence may be adversely affected by spatial aggregation error. Although spatial aggregation error has been widely investigated, few studies have examined how spatial aggregation error affects the statistical analysis of cancer data at the zip code level. This study estimates the potential impact of spatial aggregation error on the parameter values of multilevel statistical models which analyze the association between spatial accessibility to mammography facilities and late-stage breast cancer risk. This study focuses on breast cancer based on the fact that it is the most common cancer among women and an important cause of cancer mortality in Illinois [7].

This study develops a Monte Carlo simulation procedure for disaggregating cancer cases from larger to smaller study units in empirical simulations, and uses that procedure to examine the implications of spatial aggregation error for multilevel model coefficients. The context sensitivity of spatial aggregation error is also examined by comparing two study areas. This paper is divided into the following sections: literature background; data pre-processing and analytical methodology; description and analysis of results; and conclusion.

## Background

In many scientific disciplines, data are collected at a spatial scale appropriate to the research question of interest. However, in geography and public health, much data is publicly available to researchers for analysis in predefined areas (zones) with an arbitrary and modifiable boundary. These zones were not optimally designed to answer the research question, thus introducing geographical bias which affects subsequent statistical analyses based on such data. This is the well-known Modifiable Area Unit Problem (MAUP). One of the most common consequences of this problem is the ecological fallacy.

The ecological fallacy arises when making inferences from higher to lower levels of analysis [8]. The model coefficients estimated based on aggregated data differ from those at the individual level, leading to errors of interpretation [9-12]. Gehlke and Biehl (1934) found that the magnitude of the correlation coefficient increased with aggregation [9]. Openshaw and Taylor (1979) demonstrated the impact on correlation coefficients of spatial aggregation of data from smaller to larger geographic zones [12]. As in earlier work on the ecological fallacy, they found that spatial aggregation tends to increase the magnitude of correlation coefficients, confirming that spatial aggregation error has an impact on statistical analysis. Spatial aggregation error is an example of biased inference caused by the mismatch between spatial units and the research question of interest. It particularly occurs when a large area or a single point is employed to represent spatially distributed individuals [2]. Hillsman and Rhoda (1978) identified three types of the spatial aggregation error that arise when estimating a population's average distance to the nearest service facility [1]. The three types of error are based on different geographical characteristics of origins and destinations and can result in under- or over-estimation of individuals' actual travel distances.

Recently, with the rapid expansion of computational resources and GIS, spatial aggregation error has been studied more thoroughly. Researchers have adopted different approaches to evaluate the influence of spatial aggregation error in large study areas. Hewko et al., (2002) analyzed the spatial aggregation error associated with the measurement of neighborhood spatial accessibility (NSA) [3]. Neighborhood spatial accessibility describes the ease with which residents can travel to service facilities, and it can be approximated by the network distance from home to the closest facility. Because population data are typically aggregated to zones (census tracts, zip codes), distance is calculated from zonal centroids to facilities resulting in spatial aggregation error. Hewko et al. (2002) compared three methods for estimating distance: one involves the use of unweighted (geographic centroids) while the others incorporate finer-scale, block-level population data thus reducing spatial aggregation error [3]. Comparing the NSA values based on these three methods, the authors concluded that spatial aggregation error does create bias, but the impact varies with the type of centroids and the number and locations of service destinations. Spatial autocorrelation tests were also affected. Fortney, Rost & Warren (2000) studied the impact of spatial aggregation error on measures of spatial accessibility to physicians [13]. Their results showed substantial differences between area centroid-based estimates of distance to physicians and distances calculated from individual residences, confirming that spatial aggregation error leads to significant "errors in variables" in measuring spatial accessibility.

Gregorio et al., (2005) studied the impact of spatial aggregation on tests of spatial clustering. They compared the analysis of spatial clustering of late-stage cancer in Connecticut using cancer data at different geographic scales - block group, census tract and town [4]. Results showed little difference in the outcomes of spatial clustering tests using data at different scales. In this example, the impacts of spatial aggregation error were minimal, contradicting the aforementioned literature and suggesting the need for further analysis of the issue.

Examining spatial aggregation error requires the use of high resolution data; however, such high resolution data is often not available due to privacy and confidentiality restrictions [6]. Although with proper approvals, some health departments do provide access to high resolution data; in many cases it is only possible to obtain cancer data at a low spatial resolution such as county or zip code. Zip codes are devised by the U.S. Postal Service to facilitate mail delivery, and each zip code comprises a set of mail distribution points which can be joined to create zip code areas. Zip codes vary greatly in geographic and population size, with an average population size of 30000 in 2000 [14]. The large and variable sizes of zip codes, and the fact that they are not well-defined geographic zones, pose challenges for spatial analysis of health data.

Using large-area data increases the risk of spatial aggregation error. Recently, some authors have used Monte Carlo methods to analyze spatial aggregation error by assigning data from larger to smaller zones based on the demographic characteristics of individual cancer cases[5,15]. To obtain cancer data with a high resolution and reduce spatial aggregation error, Henry and Boscoe (2008) used demographically-based geo-imputation to assign cancer cases from zip codes to census tracts [15]. Cases were assigned to tracts based on their age, gender and racial characteristics, and cases were more likely to be assigned to tracts whose populations have similar demographic characteristics. To test the geographic accuracy of the assignment, the authors obtained data on the actual residential locations of cancer cases. The actual census tract of residence was compared to the tract assigned via geo-imputation. They found that the validity and reliability of the geo-imputation outcomes were dependent on demographic variables; that is, using race/ethnicity in geo-imputation provided a more accurate disaggregation than the one utilizing population only. The authors also detected that the geo-imputation performed differently within different census tracts. Homogeneous census tracts were more likely to have a low match rate than more heterogeneous ones.

Spatial aggregation error can also arise when using hybrid data with point- and polygon-levels [16-18]. Some methods of analysis require a consistent set of geographic units, so that hybrid data require conversion of data from either point to polygon or vice versa. If points are aggregated to corresponding polygons, however, localized information from point-level data is lost [19].An alternative approach is to convert polygon data to point data. For example, one can assign random locations to observations within polygons, and repeat the process many times using Monte Carlo simulation to estimate uncertainty[20]. Shi (2009) devised a restricted Monte Carlo method to assign polygon-level addresses into suitable random point locations in investigating spatial variation in lung cancer incidence in New Hampshire [21]. The method was employed to detect spatial clusters of high cancer incidence while incorporating spatial uncertainty associated with imprecise address locations. By quantifying uncertainty, this approach provides an indication of the error associated with spatial aggregation.

Although previous studies have emphasized the importance of spatial aggregation error and developed methods to reduce its effects, less is known about the impacts of spatial aggregation error on statistical estimates of model coefficients. Two recent studies investigate this issue with respect to positional error - a form of geographic error-in-variables that is similar to spatial aggregation error. Positional error occurs when residences are placed at incorrect locations due to errors in geocoding and inaccuracies in street network information. Griffith et al., (2007) studied the impacts of positional error on spatial regression analysis by comparing analytical results using datasets with different geocoding accuracies [22]. They found that positional error had a noticeable influence on parameter estimates obtained through spatial statistical analysis. Mazumdar et al., (2008) examined a similar question using somewhat different methods [23]. They found that the observed strength of association between environmental exposure and disease incidence decreased as positional error increased. The implication is that it is more difficult to uncover the true association between environmental exposures and disease using less accurate spatial data. These studies suggest that geographic error-in-variables can lead to errors in statistical estimates of model coefficients. Spatial aggregation error results in a similar kind of error-in-variables and is likely to have similar kinds of impacts on model coefficients. The only difference is that spatial aggregation error has an explicit spatial structure rooted in the zones to which data are aggregated. In contrast, positional error does not have an explicit spatial structure and can be associated with very large displacements of points from their true locations.

In this paper, we examine the impact of spatial aggregation error on the coefficients of multilevel statistical models which analyze the associations between late-stage breast cancer, demographic variables and distance to mammography facilities. Using Monte Carlo simulation methods similar to those adopted by Henry and Boscoe (2008) and Shi (2007), we generate a large number of 'disaggregations' of breast cancer cases from the zip code to the census block level [5,15]. The assignment of individual breast cancer cases from zip codes to blocks is proportional to the age/racial composition of block populations as in Henry and Boscoe (2008). We estimate a multilevel statistical model of late-stage breast cancer risk that includes a spatial variable, distance to the nearest mammography facility, as a predictor of late-stage risk. Models are estimated at the zip code and census block levels, and differences between model coefficients at the two levels reveal the impacts of spatial aggregation error.

## Methods

**Figure 1:**
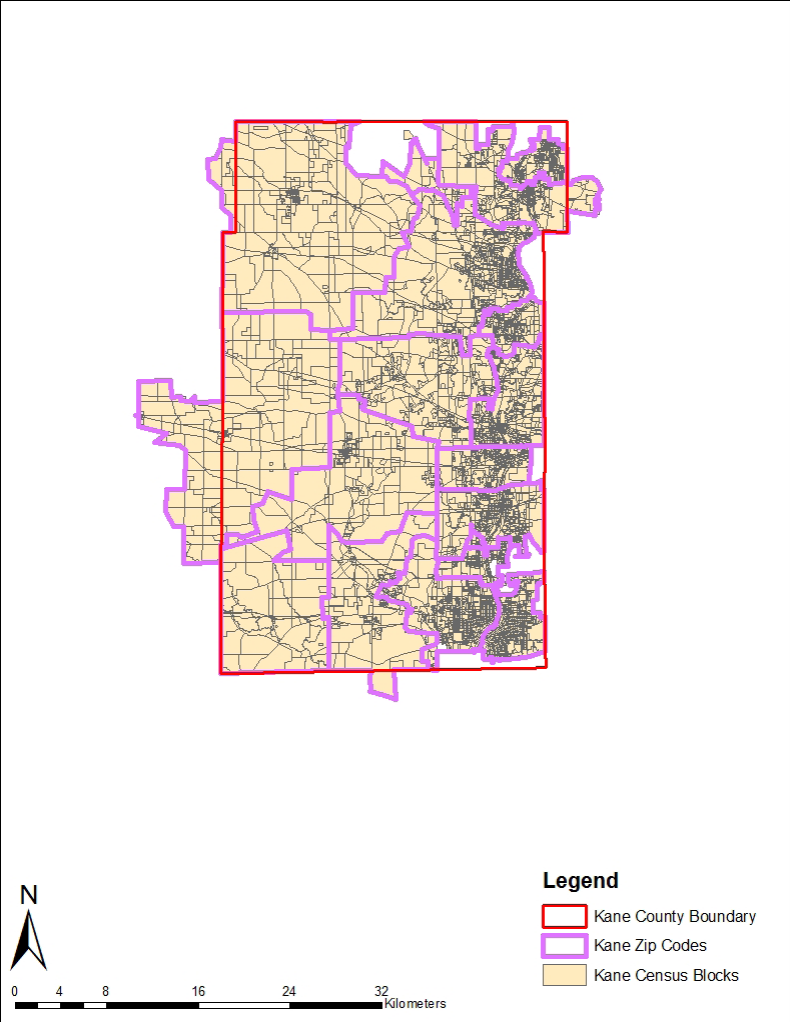
**Census Blocks and Zip Codes in the Kane County Study Area**.

**Figure 2:**
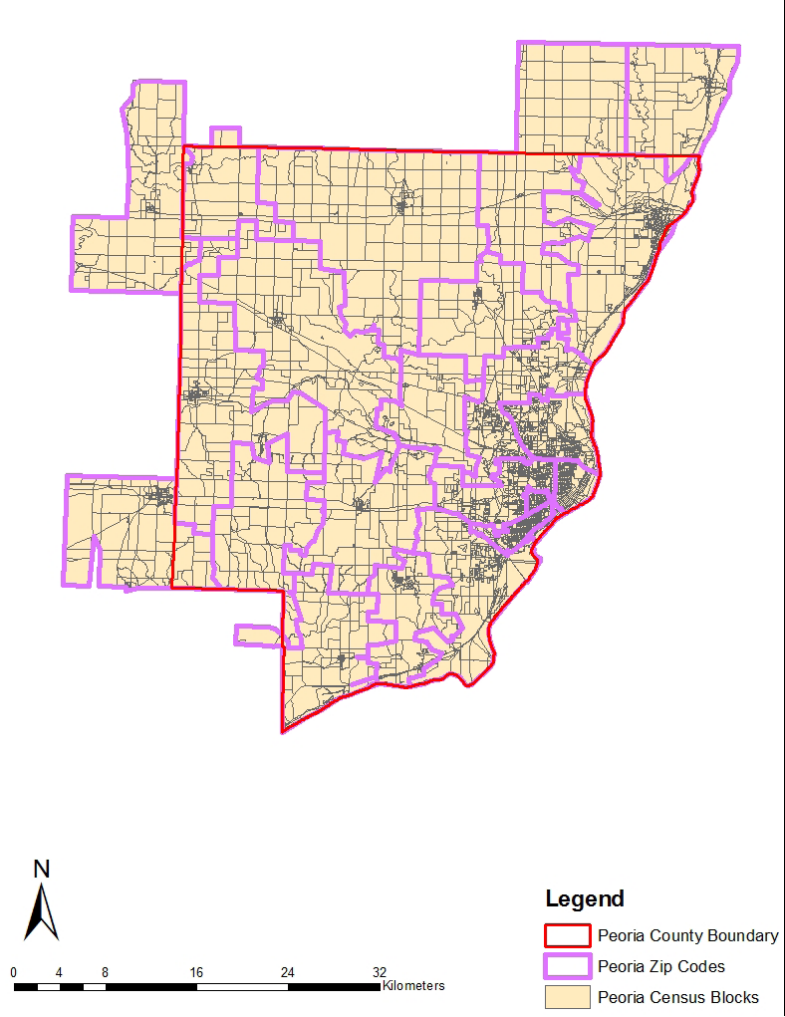
**Census Blocks and Zip Codes in the Peoria County Study Area**.

Two geographically and demographically diverse study areas are chosen for analysis: Kane and Peoria counties in Illinois. Kane County is located in the southwest section of the Chicago Metropolitan area. The eastern part of this county is highly populated, while the western part is mainly farmland with a few residential areas. The population is predominantly Caucasian, with concentrations in the young and middle age groups. African-Americans make up around 6 percent of the county's population. Peoria County is located in central Illinois. Its population characteristics are similar to those of Kane County, except for a higher representation in the elderly age group (>65 years). Because zip code boundaries sometimes cut across county borders, all contiguous zip codes are included in the study areas as long as the zip code centroids fall within county boundaries. The two study areas are illustrated in Figures 1 and 2.

Breast cancer cases in Illinois were obtained from the Illinois State Cancer Registry (ISCR). The dataset contains demographic and epidemiologic records at the individual level and each record is geocoded to the residential zip code. Variables include age group, sex, race, diagnosis stage and year. ISCR utilized a classification scheme parallel with SEER summary stage to measure cancer stage at diagnosis [24]. Cancer cases at stages 0 and 1 were considered as early stage, and cases staged from 2 to 7 were regarded as late stage [7]. Cases with unknown stage were excluded from this study. For both study areas, female breast cancer cases from 1998 to 2002 were selected. The percent of cases at different stages for the two study areas is shown in Table 1.

**Table 1:** Breast Cancer Cases by Stage in Kane and Peoria, 1998-2002

Study Area	# Cases	Unstaged		Late-Stage	
		
		# Cases	Percent (%)	# Cases	Percent (%)
Kane	1102	65	5.90	406	39.2

Peoria	804	38	4.73	245	32.0

The Monte Carlo Simulation procedure involves assigning cancer cases from a zip code area to the census blocks within that zip code. The probability of assignment is proportional to the age -race composition of the block population; so, for example, a cancer case in a black woman aged 50-69 has a higher probability of assignment to a census block that has a large population in the same demographic group. To facilitate this assignment procedure, we divided cancer cases into 6 categories based on age-race combinations. To differentiate the age categories, three age groups were used: less than 50-years old, between 50-and 70-years old, and more than 70-years old. Research shows that the risk of late-stage diagnosis varies according to age, and young patients have a higher risk of late diagnosis [25]. Cases also were divided into 'black' and 'non-black' racial categories, given that late-stage breast cancer risk is high among blacks [26-30]. The numbers of breast cancer cases in each county in the six categories are listed in Table 2.

**Table 2:** Summary of Breast Cancer Cases by Demographic Subgroup, Kane and Peoria

Kane		Peoria	
Population Subset	Cases	Population Subset	Cases

Total Population	1037	Total Population	766

Non-Black		Non-Black	

Female <50 years	243	Female <50 years	131

Female 50~70 years	420	Female 50~70 years	294

Female >70 years	334	Female >70 years	276

Black		Black	

Female <50 years	17	Female <50 years	24

Female 50~70 years	14	Female 50~70 years	30

Female >70 years	9	Female >70 years	11

Demographic information for the year 2000 at the census-block level was obtained from the U.S. Census for all the census blocks in the two study areas. There were a total of 7,619 census blocks in the Kane study area and 5,689 in Peoria. The census block female populations were divided into the six subgroups described above to match the breast cancer data.

### Shortest Travel Distance Calculation

**Figure 3:**
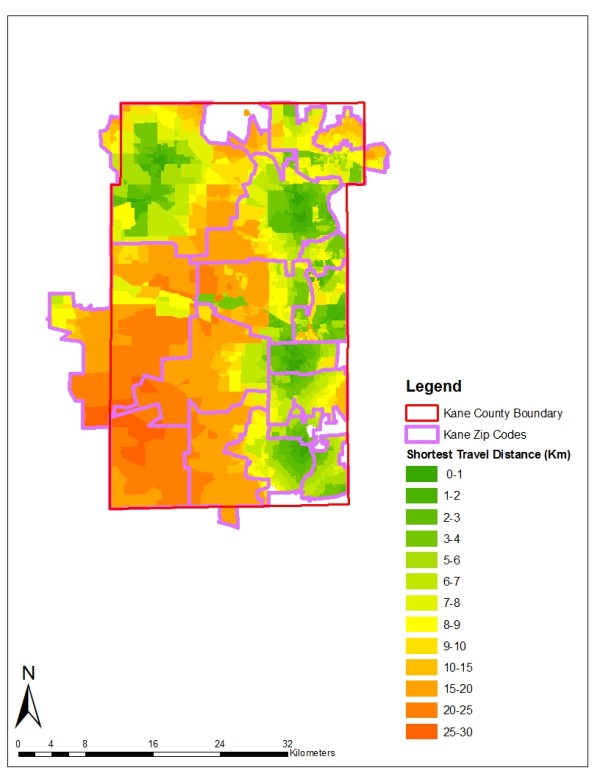
**Block-Level Shortest Travel Distance Distribution in the Kane County Study Area**.

**Figure 4:**
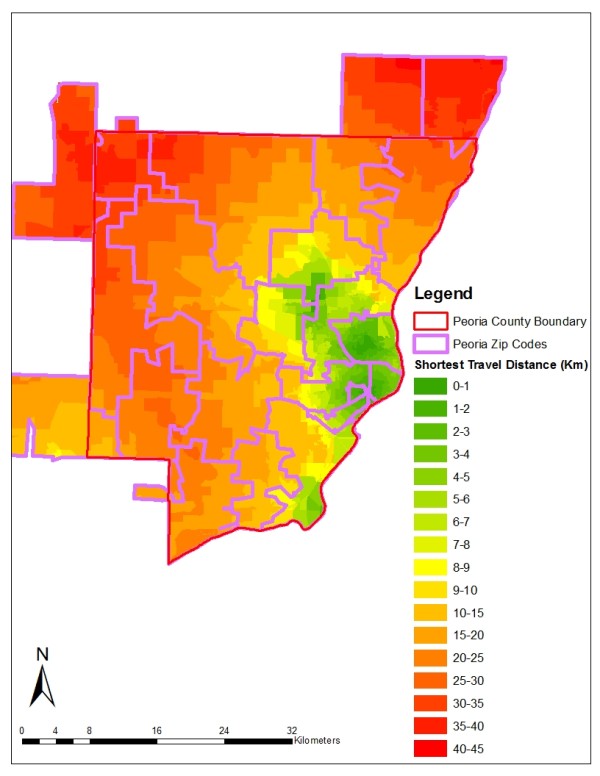
**Block-Level Shortest Travel Distance Distribution in the Peoria County Study Area**.

The spatial variable examined in this study is travel distance to the nearest mammography facility. Some research suggests that poor spatial accessibility to mammography screening facilities is associated with late-stage diagnosis. There are many ways to measure spatial accessibility, including provider-to-population ratio, and travel impedance to nearest provider [31]. We estimated spatial accessibility based on shortest travel distance --the road network distance from the zip code or block centroid to the nearest provider. Many studies have used shortest travel distance to evaluate spatial accessibility at neighborhood level [32-36]. Within each zip code, population-weighted centroids were used to better reflect the uneven distribution of population [37]. Geographic centroids were used for the block-level analysis. Data on registered mammography screening facilities in Illinois were obtained for 2000, and facilities were geocoded using street address information. Mobile mammography facilities do not operate in either county and thus were not included in the analysis. The shortest travel distance was computed from each centroid to its nearest mammography screening facility through the road network. The block-level shortest distances in the Kane and Peoria study areas are mapped in Figures 3 and 4. In both counties, the shortest distances do not exceed 46 kilometers, suggesting that spatial access to mammography is reasonably good overall.

Figures 3 and 4 show that within some zip codes, block-level travel distances vary significantly which indicates the potential for spatial aggregation error. Summary statistics for the distance variable at the zip code and block levels also reveal substantial disparities, particularly for Peoria County (Table 3). In Peoria County, the average and median travel distances differ by 4 and 9 kilometers respectively for zip codes and blocks, whereas in Kane County, the mean and median values are quite similar. This suggests that the impact of spatial aggregation error will be greater in Peoria County where the distance measurements at the two levels are very different.

**Table 3:** Summary Statistics for Travel Distance to Mammography at the Block Zip Code Levels

Variables(Km)		Kane Study Area		
	
	Min	Max	Mean	Median
Block-level Distance	0.0315	22.601	5.951	4.995

Zip-level Distance	0.670	13.149	5.621	4.564

**Variables(Km)**		**Peoria Study Area**		
	
	**Min**	**Max**	**Mean**	**Median**

Block-level Distance	0.0527	43.255	11.902	8.027

Zip-level Distance	1.373	36.262	15.569	17.110

### Disaggregation of Breast Cancer Data Using Monte Carlo Simulation

The purpose of the Monte Carlo simulation is to investigate the impact of the spatial aggregation error by comparing zip code-level model coefficient estimates with a reference distribution of values based on small-area (block level) data. Ideally, one would want to compare the zip-code values with those based on actual patient residential locations. However, because of privacy and confidentiality issues, we were unable to obtain breast cancer data below the zip code level. Therefore, simulation was used to create 'reasonably' distributed cancer cases at the census block level, building upon the work of [5] and [15]. Each case was randomly assigned to a block within its zip code, and the likelihood of assignment depended on the age-race composition of the block population defined according to the six subgroups mentioned earlier.

To implement the Monte Carlo simulation, the block-level population in each demographic subgroup was accumulated and summed. The output was then normalized so that each subgroup's population ranged from 0 to 1, with intermediate values representing the cumulative share of that subgroup's population located in each block. This process was repeated for each zip code and each subgroup. Based on this data, the Monte Carlo simulation was implemented.

The Monte Carlo simulation involved several steps. First, for each cancer case, an array of 1,000 uniform random numbers was generated. A nested-structure of generating seeds was used to ensure the independence of each random number. Specifically, a series of random numbers was generated using system time as the generating seed. Then this series was employed as the secondary generating seed to produce final numbers. The end result was an 'n' by 1000 matrix in which n is the number of cancer cases. Rows represent individual cancer cases and columns represent random numbers. Second, we used the random numbers to assign a case from zip code to a block, with each random number representing a simulated block assignment. Each block assignment was based on the following principle: a case was assigned to a census block if the block-level normalized range of values contained that specific random number. Hence, the assignment was not based on a uniform distribution, but was proportional to the block population falling in the same demographic category as the cancer case. Assignments were made sequentially within each column of the matrix. Third, within a specific column, once a block received a cancer case, the block population in that demographic category was reduced by 1, because one person cannot be diagnosed with cancer twice simultaneously. If a population subgroup within a block went down to zero, the block was taken out from the remaining candidates for subsequent assignments in the same demographic category. We iterated the second and third steps, disaggregating cases from zip codes to blocks, and thus generated 1,000 disaggregated patterns of cases. As a result, a final matrix was produced in which rows represented cancer cases and columns denoted different assignments of census blocks for each cancer case. The matrix was diagramed as 1,037 rows by 1,000 columns for cases in Kane, and 766 rows by 1,000 columns for cases in Peoria. We wrote the Monte Carlo simulation procedure using Javascript1.5 and used Eclipse 3.4.0 as the software interface.

### Analysis of Spatial Aggregation Error Using Hierarchical Logistic Regression

Hierarchical (multilevel) logistic regression was utilized to evaluate the impact of spatial aggregation error on statistical models of late-stage breast cancer risk. We used a two-level hierarchical modeling approach in which individual cancer patients (level 1) are nested within either zip codes or blocks (level 2). First, hierarchical models were estimated with the zip code as level 2; then, after Monte Carlo simulation, models were estimated at the block scale, with blocks representing level 2. The dependent variable in the hierarchical regression models is late-stage diagnosis. Only a limited set of independent variables is included in the models so that the effects of spatial aggregation error can easily be observed. Individual variables include the patient's age and race categories, defined according to the categories used earlier. Race is represented by a dummy variable (BLACK) in which 'non-black' is the reference category. Age is represented by two dummy variables (AGE < 50, AGE 50-70), and the reference category is the oldest age group (> = 70). The level 2 independent variable is shortest travel distance (in meters) to the closest mammography facility, measured based on zip code centroid for the zip code model and block centroid for the block-level disaggregations. The formulations of the hierarchical logistic regression are shown below:

The micro specification (level 1) is:(1)

where the *β*s denote the constant (intercept) and regression coefficients of the independent variables, *i *= 1,..., n_j _denotes individuals within different zip code or census block areas, and *j *= 1,..., J denotes zip code or census block areas. The *R*_*ij *_are micro errors with independent normal distributions, *R*_*ij *_~*N*(0, *σ*^*2*^).

The macro stage (level 2) model is:(2)

where *Us *are macro errors, *U*_*oj *_~*N*(0,) and they are independent over *j *and with *R*_*ij*_. Equations (1.1) and (1.2) define a hierarchical logistic model that can be written equivalently as a combined single-equation model by substituting (1.1) - (1.2) into (2):(3)

The variable most likely to be affected by spatial aggregation error is shortest travel distance, so any change in model coefficients between the zip code and block levels is mainly due to changes in measurement of this variable resulting from spatial aggregation.

All models were estimated using 'proc glimmix' in SAS 9.1 [38]. Given that there are 1,000 randomized patterns of cases at the block level, macro-level SAS syntax was used to automatically estimate the block-level hierarchical regression analyses. The coefficient estimates for the block level models were displayed as histograms and compared with the respective values for the zip code level coefficients.

## Results

**Figure 5:**
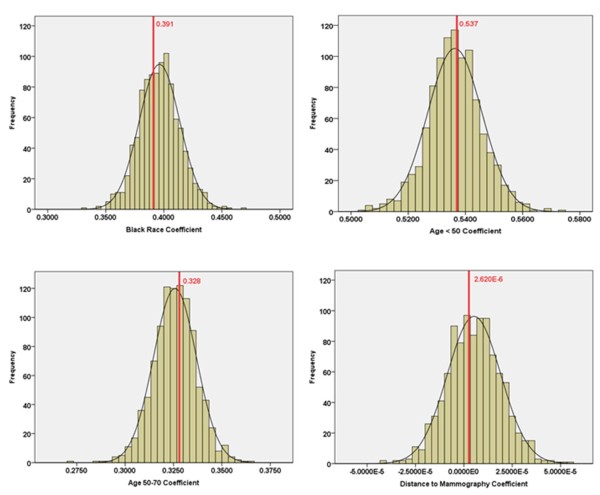
**Zip code-level coefficient (red, bold line) and histogram of block-level coefficients for each independent variable, Kane County**.

The comparison of model coefficients at the zip code and block levels for Kane County is shown in Table 4 and Figure 5. The results for Kane show only a small impact of spatial aggregation error on model coefficients. The means of the block coefficients are very close to the corresponding zip code values except in the case of shortest travel distance. In addition, the ranges of block-level coefficients include the corresponding zip code parameters for all independent variables. The similarity of zip code and block level coefficients is also evident in Figure 5 which shows, for each independent variable, a histogram of the block-level coefficients and a dotted line representing the zip code coefficient. All of the zip code coefficients are located near the peak of their corresponding block-level histograms. Moreover, at both levels, the coefficient for distance indicates no statistically significant association between shortest travel distance to mammography and late-stage diagnosis, so the overall findings are consistent. Therefore, for the Kane study area, the closeness of the means and the fact that the zip code values fall within their respective block-level ranges show that spatial aggregation error does not have much influence on inferences made based on statistical analysis at the zip code level.

**Table 4:** Model Coefficients at the Block and Zip Code Levels for Kane County

Variables	Census Block Level			Zip Code Level			
	
	Mean Coefficient	Min	Max	Coefficient	Std Error	p-value	95% Confidence Interval
Age < 50	0.536	0.503	0.575	0.537	0.171	0.002	(0.201, 0.873)

Age 50~70	0.326	0.273	0.365	0.328	0.152	0.031	(0.0305, 0.626)

Black	0.396	0.332	0.468	0.391	0.326	0.230	(-0.248, 1.031)

Shortest Travel Distance (m)	5.270E-6	-4.155E-5	5.394E-5	2.620E-6	2.489E-5	0.916	(-4.623E-5, 5.147E-5)

The findings are very different for the Peoria study area. Large differences are evident between zip code- and block- coefficients. As shown in Tables 5, each of the zip code-level coefficients falls outside the range of the respective block-level coefficients. Also, the zip code coefficients differ much more from their respective block means than was the case in the Kane study area. This is especially true for shortest travel distance, in which the coefficient signs for models at the two levels are different. Specifically, for shortest travel distance, the zip code-level parameter is negative and an order of magnitude less (in the negative direction) than the mean of the block-level values which has a positive sign.

**Table 5:** Model Coefficients at the Block and Zip Code Levels for Peoria County

Variables	Census Block Level			Zip Code Level			
	
	Mean Coefficient	Min	Max	Coefficient	Std Error	p-value	95% Confidence Interval
Age < 50	0.673	0.661	0.683	0.714	0.219	0.0012	(0.283, 1.145)

Age 50~70	0.445	0.434	0.454	0.482	0.184	0.0089	(0.121, 0.842)

Black	1.082	1.040	1.128	0.943	0.271	0.0005	(0.411, 1.475)

Shortest Travel Distance (m)	1.419E-5	5.950E-6	2.250E-5	-3.51 E-4	2.08 E-4	0.091	(-7.596E-4, 5.678E-5)

For the Peoria case, we calculated the impact of these differences in model coefficients on model predictions by plugging in values for a "reference person" (non-black, age >70) located at distances of 0 and 20 km from the closest mammography facility. At zero kilometers, the predicted late-stage risks are very similar for the zip code and block (average) models -- 0.250 and 0.266 respectively. However, at 20 kilometers, differences are extraordinarily large because the effects of the different distance coefficients are magnified. The zip code model gives a predicted late-stage risk of less than 1 percent, a nonsensical value; whereas the block (average) model yields a predicted risk of 24 percent. Thus, using the zip code model for predictive purposes does not give meaningful results.

**Figure 6:**
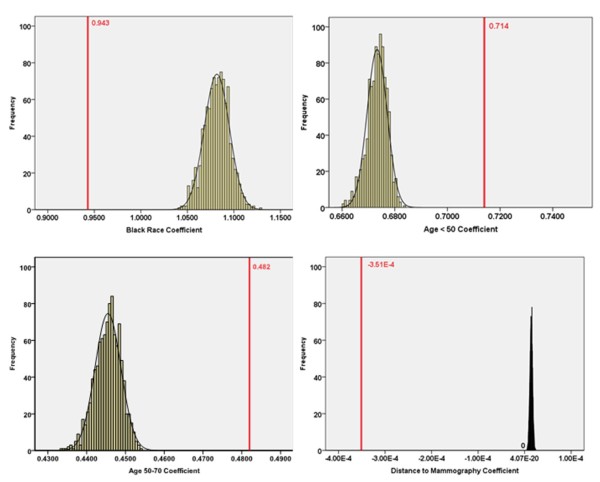
**Zip code-level coefficient (red, bold line) and histogram of block-level coefficients for each independent variable, Peoria County**.

For Peoria County, impacts of spatial aggregation error are also apparent in the plots comparing model coefficients at the zip code and block levels (Figure 6). For each independent variable, the zip code-level coefficient falls substantially outside the range of the block-level values. For the distance variable, the zip code level parameter estimate is completely isolated from the block-level values, differing greatly in magnitude and with the opposite sign as noted above. This suggests that the association between distance and late-stage cancer risk is completely different from that observed based on zip code data. Among the remaining demographic variables, the coefficient for black race changed more than those for the two age variables. Coefficients for the two age variables move towards zero when we shift from the zip code to block scale, whereas the coefficient for black race increases. Block-level models indicate that black race is more strongly associated with late-stage breast cancer risk than was evident in the zip code-level model. Thus, spatial aggregation error affects not only the coefficient for the spatial variable in the model, distance to mammography, but also the coefficients for the other socio-demographic variables, age and race, which were incorporated in the Monte Carlo simulation procedure.

## Discussion

These results indicate that in some geographic contexts, spatial aggregation error results in significant bias in model coefficients, bias that can lead to inaccurate conclusions and inappropriate statistical inferences. Results for Peoria County suggest that if cancer data were available at the block level, the resulting model coefficients for all independent variables would most likely be quite different from the values observed based on zip code data. For the distance variable, the impact of spatial aggregation error is substantial enough to affect statistical inference. In particular, a significance test (one-sided, α = 0.1) indicates that the zip code-level coefficient for the distance variable is significantly different from zero, suggesting that distance to mammography is significantly and negatively associated with late-stage breast cancer risk. This is an unexpected finding implying that late-stage risk decreases with increasing distance,. Yet our simulations indicate that this conclusion is most likely a spurious result of spatial aggregation error. The block-level coefficients are all close to zero, suggesting a lack of statistical association. Without address-level data, we cannot know the true association between distance and late-stage breast cancer risk; however, the simulated block-level values overwhelmingly suggest no association.

Although spatial aggregation error is important, the differences between the two study areas reveal that the influence of spatial aggregation error is highly case-sensitive. In Kane County, spatial aggregation has a minimal impact on model coefficients; whereas in Peoria County, the impact is substantial. We believe that these differences are linked to differences in the underlying spatial distributions of socio-demographic groups and differences in the sizes and configurations of zip codes and blocks which are superimposed on each county's demographic landscape. We can only speculate about the causes of differences observed between these two counties. Located on the fringe of the Chicago metropolitan region, Kane County has a higher population density than Peoria County, and Kane's population appears to be more uniformly distributed, although with an east-west gradient. Mammography facilities are well-distributed throughout the more populated areas of the county. In comparison, Peoria County contains a more bifurcated rural-urban pattern, with a single, densely populated city (Peoria) surrounded by low density suburban and rural zones. The few mammography facilities are concentrated in Peoria city. In this bifurcated landscape, disaggregation of cases to the block level via Monte Carlo simulation results in heterogeneous assignments that greatly influence model coefficients.

Another important finding is that the statistical impacts of spatial aggregation error are not confined to coefficients for spatial variables. In Peoria County, coefficients for all variables are affected. These interconnected impacts most likely reflect the correlations between race, age and residential location. Residential segregation by race is a strong feature of both study areas, and it implies that the 'black' and 'non-black' racial categories have distinct residential geographies at the block scale. Disaggregating data from zip codes to blocks on the basis of racially- and demographically-based probabilities incorporates these localized, segregated geographies. Although we used population-weighted centroids in calculating shortest distance to mammography, using race- and age-specific population centroids may be more effective in minimizing spatial aggregation error associated with residential segregation. Still, these more finely-tuned centroids can be problematic when racial groups are both segregated and unevenly distributed within zip code boundaries as is often the case.

## Conclusion

The paper analyzed the impact of the spatial aggregation error on zip code level statistical analysis of the associations between spatial and non-spatial variables and late-stage breast cancer risk in two study areas in Illinois. Given the difficulties in obtaining cancer cases below the zip code level, we designed a Monte Carlo simulation procedure to disaggregate cancer cases from zip codes to census blocks on the basis of the demographic characteristics of cancer case and block populations. Spatial aggregation error significantly affected the coefficients of statistical models in the Peoria study area, leading to inaccurate inference, whereas in Kane County the impact was minimal. The distinctive outputs for Kane and Peoria counties illustrate that the impacts of spatial aggregation error are context-dependent. Impacts appear to be most pronounced in areas like Peoria County, which have both highly uneven and segregated residential geographies. The spatial autocorrelation of age- and racially-categorized population groups by block may be important in affecting spatial aggregation error. Error also depends on the configuration of zones overlaying those geographies. Many studies have demonstrated that large zones are associated with high levels of spatial aggregation error, but clearly the residential geographies within the zones are also important. Other factors affecting spatial aggregation error in analyzing distance to health services are the number and spatial configuration of service facilities [3]. In general, the potential for error will be greater in places with fewer facilities and where facilities are spatially clustered. Compared to Peoria, Kane County has more mammography facilities, and facilities are more spatially dispersed, perhaps reducing the scope for spatial aggregation error.

Given the range and complexity of factors involved in spatial aggregation error, the specific nature of these associations requires further investigation using a much wider range of study areas representing varied social and geographical characteristics. The Monte Carlo simulation procedure implemented here is very useful in these efforts. Moreover, analyzing how spatial aggregation error compares with other kinds of uncertainty such as sampling error in statistical modeling is also critically important.

Our findings highlight the need to develop methods and procedures for minimizing spatial aggregation error in statistical models that rely on zonal health data. Monte Carlo simulation provides a way to generate the highly likely distribution of block-level coefficients associated with a particular dataset, but the method is both data- and computationally-intensive. Much simpler procedures, like using age- and race-specific zip code centroids offer a feasible, low-tech alternative, but these methods may not be effective in areas where the spatial distributions of population groups are highly uneven [39]. Shi and Berke (2009) discuss promising methods which utilize area-based representations of population [40]. Another option is explicit modeling of aggregation effects through the use of variograms and other indicators of spatial autocorrelation. Promising methods have been developed for use with environmental and population data [41], and the methods have great potential value for health studies [42].

Although we have demonstrated the importance of spatial aggregation error, our study has several limitations. Because we do not have access to data on actual breast cancer cases locations, we do not know the real extent and impact of spatial aggregation error in the two case study areas. The simulations delineate the likely distribution of possible coefficient values, but do not quantify the true spatial aggregation error. Still knowing the likely extent of error is important in signaling the need for more advanced methodologies that explicitly address spatial aggregation effects. Another limitation is that by relying on actual cancer case data we have no knowledge of, the underlying 'true' risk model for late-stage breast cancer, and we are unable to control or manipulate that model in the process of Monte Carlo simulation. A more experimental approach based on hypothetical data would enable researchers to assess the relative magnitude of spatial aggregation error compared to other sources of error in statistical models of cancer risk factors. Despite these limitations, this research demonstrates that spatial aggregation error has substantial effects in some geographic contexts on the results of statistical modeling of the association between cancer and spatial and non-spatial risk factors. Understanding how and why these effects vary stands as a key topic for future research investigations.

## Competing interests

The authors declare that they have no competing interests.

## Authors' contributions

LL planned, coded and implemented the simulation model, performed the statistical analyses and assisted in drafting the manuscript. SM conceived the study, designed the methodology, supervised the analyses and drafted the manuscript. FW acquired the data and assisted in drafting the manuscript. All authors read and approved the final manuscript.

## References

[B1] Hillsman E Rhoda R Errors in Measuring Distances from Populations to Services Centers Annals of Regional Science 1978 12 74 88 10.1007/BF01286124

[B2] Hodgson MJ Shmulevitz F Kőrkel M Aggregation Error Effects on the Discrete-Space *P*-Median Model: The Case of Edmonton, Canada The Canadian Geographer 1997 41 415 428 10.1111/j.1541-0064.1997.tb01324.x

[B3] Hewko J Smoyer-Tomic KE Hodgson MJ Measuring Neighborhood Spatial Accessibility to Urban Amenities: Does Aggregation Error Matter? Environment and Planning A 2002 34 1185 1206 10.1068/a34171

[B4] Gregorio D DeChello L Samociuk H Kulldorff M Lumping or Splitting: Seeking the Preferred Areal Unit for Health Geography Studies International Journal of Health Geographics 2005 4 6 10.1186/1476-072X-4-6 15788100 PMC1079921

[B5] Shi X Evaluating the Uncertainty Caused by Post Office Box Addresses in Environmental Health Studies: A Restricted Monte Carlo Approach International Journal of Geographical Information Science 2007 21 3 325 340 10.1080/13658810600924211

[B6] Rushton G Armstrong P Gittler J Greene B Pavlik CE West MM Zimmerman DL Geocoding in Cancer Research A Review American Journal of Preventive Medicine 2006 30 25 16 24 10.1016/j.amepre.2005.09.011 16458786

[B7] Wang F Luo L McLafferty S Health Access, Socioeconomic Factors and Late-Stage Cancer Diagnosis: An Exploratory Spatial Analysis and Public Policy Implication International Journal of Public Policy 2010 5 2/3 237 258 10.1504/IJPP.2010.030606 PMC3540777 23316251

[B8] Johnston RJ Johnston RJ, Gregory D, Pratt G, Watts M Ecological Fallacy The Dictionary of Human Geography 2000 Oxford: Blackwell 190 191

[B9] Gehlke CE Biehl H Certain Effects of Grouping upon the Size of the Correlation Coefficient in Census Tract Material Journal of the American Statistical Association, Supplement 1934 29 169 170 10.2307/2277827

[B10] Yule GU Kendall MG An Introduction to the Theory of Statistics Griffin 1950 London

[B11] Robinson AH Ecological Correlation and the Behavior of Individuals American Sociological Review 1950 15 351 357 10.2307/2087176

[B12] Openshaw S Taylor PJ Wrigley N A Million or so Correlation Coefficients: Three Experiments on the Modifiable Areal Unit Problem Statistical Methods in the Spatial Science 1979 London 127 144

[B13] Fortney J Kathryn R Warren J Comparing Alternative Methods of Measuring Geographic Access to Health Services Health Services & Outcomes Research Methodology 2000 1 2 173 184

[B14] Krieger N Waterman P Chen J Soobader M Subramanian S Carson R Zip Code Caveat: Bias Due to Spatiotemporal Mismatches Between Zip Codes and US Census-Defined Geographic Areas - The Public Health Disparities Geocoding Project American Journal of Public Health 2002 92 1100 1102 10.2105/AJPH.92.7.1100 12084688 PMC1447194

[B15] Henry KA Boscoe FP Estimating the Accuracy of Geographical Imputation International Journal of Health Geographics 2008 7 3 1 10 18215308 10.1186/1476-072X-7-3 PMC2266732

[B16] Krieger N Waterman P Lemieux K Zierler S Hogan JW On the Wrong Side of the Tracts? Evaluating the Accuracy of Geocoding in Public Health Research American Journal of Public Health 2001 91 1114 1116 10.2105/AJPH.91.7.1114 11441740 PMC1446703

[B17] Bonner M Han D Nie J Rogerson P Vena J Freudenheim J Positional Accuracy of Geocoded Addresses in Epidemiologic Research Epidemiology 2003 14 408 412 12843763 10.1097/01.EDE.0000073121.63254.c5

[B18] McElroy JL Remington P Trentham-Dietz A Robert SA Newcomb PA Geocoding Addresses from A Large Population-Based Study: Lessons Learned Epidemiology 2003 14 399 407 12843762 10.1097/01.EDE.0000073160.79633.c1

[B19] Jacquez GM Waller LA Mowrer HT, Congalton RG The Effect of Uncertain Locations on Disease Cluster Statistics. In Quantifying Spatial Uncertainty in Natural Resources: Theory and Applications for GIS and Remote Sensing Chelsea MI: Sleeping Bear Press 1999 53 64

[B20] Jacquez GM Jacquez JA Lawson A, Biggeri D, Bőhning E, Lesaffre J-F V, Bertollini R Disease Clustering for Uncertain Locations Disease Mapping and Risk Assessment for Public Health Decision Making 1999 Wiley, London 151 168

[B21] Shi X A Geocomputational Process for Characterizing the Spatial Pattern of Lung Cancer Incidence in New Hampshire Annals of the Association of American Geographers 2009 99 3 521 533 10.1080/00045600902931801

[B22] Griffth DA Millones M Vincent M Johnson DL Hunt A Impacts of Positional Error on Spatial Regression Analysis: A Case Study of Address Locations in Syracuse, New York Transactions in GIS 2007 11 5 655 679 10.1111/j.1467-9671.2007.01067.x

[B23] Mazumdar S Rushton G Smith BJ Zimmerman DL Donham KJ Geocoding Accuracy and the Recovery of Relationships between Environmental Exposures and Health International Journal of Health Geographics 2008 7 13 1 18 18387189 10.1186/1476-072X-7-13 PMC2359739

[B24] Young JL Roffers SD Ries LAG Fritz AG Hurlbut AA (eds) SEER Summary Staging Manual-2000: Codes and Coding Instructions 2001 Bethesda MD: National Cancer Institute, NIH Pub No. 01-4969

[B25] Joslyn SA Foote ML Nasseri K Coughlin SS Howe HL Racial and Ethnic Disparities in Breast Cancer Rates by Age: NAACCR Breast Cancer Project Breast Cancer Research and Treatment 2005 92 2 97 105 10.1007/s10549-005-2112-y 15986118

[B26] Hunter CP Redmond CK Chen VW Austin DF Greenberg RS Correa P Muss HB Forman MR Wesley MN Blacklow RS Breast Cancer: Factors Associated with Stage at Diagnosis in Black and White Women. Black/White Cancer Survival Study Group Journal of the National Cancer Institute 1993 85 14 1129 1137 10.1093/jnci/85.14.1129 8320742

[B27] Eley JW Holly AH Chen VW Austin DF Wesley MN Muss HB Greenberg RS Coates RJ Correa P Redmond CK Hunter CP Herman AA Kurman R Black R Shapiro S Edwards BK Racial Differences in Survival From Breast Cancer Results of the National Cancer Institute Black/White Cancer Survival Study Journal of the American Medical Association 1994 272 12 947 954 10.1001/jama.272.12.947 8084062

[B28] McCarthy EP Burns RB Coughlin SS Freund KM Rice J Marwill SL Ash A Shwartz A Moskowitz MA Mammography Use Helps To Explain Differences in Breast Cancer Stage at Diagnosis between Older Black and White Women Annals of Internal Medicine 1998 128 9 729 736 9556466 10.7326/0003-4819-128-9-199805010-00005

[B29] Yost K Perkins C Cohen R Morris C Wright W Socioeconomic Status and Breast Cancer Incidence in California for Different Race/Ethnic Groups Cancer Causes and Control 2001 12 8 703 711 10.1023/A:1011240019516 11562110

[B30] Lannin DR Mathews HF Mitchell J Swanson MS Impacting Cultural Attitudes in African-American Women to Decrease Breast Cancer Mortality The American Journal of Surgery 2002 184 5 418 423 10.1016/S0002-9610(02)01009-7 12433605

[B31] Guagliardo MF Spatial Accessibility of Primary Care: Concepts, Methods and Challenges International Journal of Health Geographics 2004 3 3 1 13 14987337 10.1186/1476-072X-3-3 PMC394340

[B32] Athas W Adams-Cameron M Hunt W Amir-Fazli A Key C Travel Distance to Radiation Therapy and Receipt of Radio-Therapy Following Breast-Conserving Surgery Journal of the National Cancer Institute 2000 92 269 271 10.1093/jnci/92.3.269 10655446

[B33] Hyndman JCG Holman CFJ Dawes VP Effects of Distance and Social Disadvantage on the Response to Invitations to Attend Mammography Screening Journal of Medical Screening 2000 7 3 141 145 10.1136/jms.7.3.141 11126163

[B34] Nattinger AB Kneusel RT Hoffmann RG Gilligan MA Relationhsip of Distance from A Radiography Facility and Initial Breast Cancer Treatment Journal of the National Cancer Institute 2001 93 17 1344 1346 10.1093/jnci/93.17.1344 11535710

[B35] Maheswaran R Pearson T Jordan H Black D Socioeconomic Deprivation, Travel Distance, Location of Sevice, and Uptake of Breast Cancer Screening in North Derbyshire, UK Journal of Epidemiology and Community Health 2006 60 208 212 10.1136/jech.200X.038398 16476749 PMC2465550

[B36] Chen AY Halpern MT Schrag NM Stewart A Leitch M Ward E Disparities and Trends in Sentinel Lymph Node Biopsy Among Early-Stage Breast Cancer Patients (1998-2005) Journal of the National Cancer Institute 2008 100 7 462 474 10.1093/jnci/djn057 18364506

[B37] Wang F McLafferty S Escamilla V Luo L Late-Stage Breast Cancer Diagnosis and Health Access in Illinois The Professional Geographer 2008 60 54 69 10.1080/00330120701724087 18458760 PMC2367325

[B38] Statistical Analysis Software Proc Glimmix Procedure SAS Institute 2007 Cary, North Carolina http://support.sas.com/rnd/app/da/glimmix.html

[B39] Langford M Higgs G Measuring potential access to primary healthcare services: The influence of alternative spatial representations of population Professional Geographer 2006 58 3 294 306 10.1111/j.1467-9272.2006.00569.x

[B40] Shi X Berke E Computing travel time when the exact address is unknown: A comparison of point and polygon ZIP code approximation methods International Journal of Health Geographics 2009 8 23 10.1186/1476-072X-8-23 19400969 PMC2683820

[B41] Kyriakidis P A Geostatistical Framework for Area-to-Point Spatial Interpolation Geographical Analysis 2004 36 3 259 289 10.1353/geo.2004.0009

[B42] Goovaerts P Medical Geography: A Promising Field of Application for Geostatistics Mathematical Geosciences 2009 41 243 264 10.1007/s11004-008-9211-3 PMC2675168 19412347

